# Preponderance of the oncogenic V599E and V599K mutations in *B-raf *kinase domain is enhanced in melanoma cutaneous/subcutaneous metastases

**DOI:** 10.1186/1471-2407-5-58

**Published:** 2005-06-03

**Authors:** Martin Deichmann, Marianne Thome, Axel Benner, Martin Kirschner, Judith Hassanzadeh, Hjalmar Kurzen

**Affiliations:** 1 Department of Dermatology, Heidelberg University Clinics, Voßbstraße 2, 69115 Heidelberg, Germany; 2 Central Unit of Biostatistics, German Cancer Research Center, Im Neuenheimer Feld 280, 69120 Heidelberg, Germany; 3 Department of Dermatology, Venereology and Allergology, University Medical Center Mannheim, Ruprecht-Karls University of Heidelberg, Theodor-Kutzner-Ufer 1–3, 68135 Mannheim, Germany

## Abstract

**Background:**

Downstream of Ras, the serine/threonine kinase B-raf has been reported to be mutated, among other carcinomas, in a substantial subset of primary melanomas with a preponderance of mutations within the kinase domain including the activating V599E and V599K transitions.

**Methods:**

We here investigated a representative series of 60 resection specimens of cutaneous and subcutaneous melanoma metastases for the presence of mutations within the activation segment (exon 15) of the *B-raf *kinase domain by polymerase chain reaction (PCR) and single-strand conformation polymorphism (SSCP) gel electrophoresis.

**Results:**

Sequencing of cloned PCR-SSCP amplicons resulted in 24 (40%) samples harbouring somatic mutations which is not exceeding the mutation frequency in recently investigated primary melanomas. The activating mutation T1796A was present in 24/60 (40%) resection specimens, followed in frequency by the oncogenic g1795A mutation in 8/60 (13%) cases. As to the B-raf protein sequence, the acidic amino acid transitions V599E and V599K were predicted in 19/60 (32%) and 6/60 (10%) cases, resepectively, but were not associated with enhanced risk for subsequent metastasis in patients' follow up. In comparison to the primary melanomas that we recently investigated, the spectrum of predicted B-raf protein mutations narrowed significantly in the cutaneous/subcutaneous metastases. Unexpectedly, V599 and V599E mutations were absent in cutaneous/subcutaneous metastases derived from acrolentiginous melanomas as preceding primary tumours.

**Conclusion:**

During transition from primary melanomas towards cutaneous/subcutaneous metastases, the spectrum of predicted B-raf mutations narrows significantly. Focusing on the V599E and V599K, these oncogenic mutations are likely to affect melanocyte-specific pathways controlling proliferation and differentiation.

## Background

Melanomas are one of the most aggressive of the skin cancers and have shown a dramatic increase in both incidence and mortality over the past decades [1]. Several genes implicated in the development of various malignancies, among them the tumour suppressor genes *p53*, *CDKN2A *and *PTEN *and the *Ras *oncogenes, have been extensively studied in melanoma and found to be rarely mutated in resection specimens [2]. Recently, the *B-raf *oncogene has been reported to be mutated, among other carcinomas, in a majority of melanoma cell lines [3,4]. Remarkably, all mutations were within the kinase domain, with a single amino acid substitution (V599E) accounting for 95% of *B-raf *mutations in melanoma cell lines [3,4] and leading to constitutive kinase activity [3]. In preliminary series of primary melanomas, 5/9 [3] and 4/5 [5] of the resection specimens harboured this V599E mutation, and only a single different mutation occurred [3]. Investigating a representative series of 50 resection specimens of primary cutaneous melanomas, we detected 12/50 cases (24%) to harbour this V599E transition [6].

As limited attention has been given to metastatic melanoma clinical specimens yet [7,8], we decided to analyse a representative number of resection specimens for the presence of mutations in the activation segment (exon 15) of the *B-raf *kinase domain in cutaneous/subcutaneous melanoma metastases. Applying polymerase chain reaction (PCR) and single-strand conformation polymorphism (SSCP) gel electrophoresis, followed by DNA cloning and sequencing, we here describe *B-raf *kinase domain mutations in a substantial subset of cutaneous/subcutaneous melanoma metastases, the oncogenic T1796A substitution being the most frequent followed by the neighboured g1795A transition.

## Methods

### Isolation of genomic DNA from paraffin embedded tissues

Total cellular DNA was extracted from paraffin embedded tissues of 60 melanoma cutaneous and subcutaneous metastases from 60 different patients from which melanoma primary tumours of the skin had been excised (previous primary tumours: 16 superficial spreading melanomas, 8 nodular melanomas, 8 acrolentiginous melanomas, 4 lentiginous melanoma, 18 melanomas not further classified, 4 no specification, 2 no primary melanoma). Using archival tissues for this study from previous patients' therapeutic surgery, there were no objections raised by the local ethics committee. DNA was isolated from both tumour and surrounding skin tissues by microdissection. Slides with 15 μm tissue sections were incubated in xylene for 30 min and in a series of 100%, 80%, 60%, 40% ethanol and in aqua, 10 s each, at room temperature. Tissue was scratched from the slides under microscope, put in tubes, and isolation of DNA was performed using the QIAamp DNA Mini Kit (Qiagen, Valencia, CA, USA) according to the manufacturer's instructions. The quality of the isolated DNA was assessed by agarose gel electrophoresis.

### Polymerase chain reaction (PCR)

Prior to PCR-SSCP analysis of *B-raf *exon 15 in melanomas, a 184 base pair (bp) fragment of the human *A-myb gene *was amplified by primers A-Myb-1 and A-Myb-2 to ensure the DNA integrity of the genomic DNA samples as well as the absence of major Taq polymerase inhibitors. PCR was performed in a final volume of 25 μl containing 0.6 units proofreading FailSafe Taq DNA polymerase and 12.5 μl FailSafe PCR 2X PreMix F (Biozym, Oldendorf, Germany), containing 100 mM Tris-HCl pH 8.3, 100 mM KCl, 400 μM of each dNTP, supplemented by 0.25 μl of each primer (100 pmol/μl). PCR conditions were: 95°C 2 min, 46 cycles of 95°C 30 sec, 58°C 30 sec, 72°C 30 sec, followed by 72°C 7 min. All PCRs were performed on a GeneAmp 2400 thermal cycler (Perkin Elmer, Norwalk, CT, USA). PCR products were analysed by 1.5% agarose gel electrophoresis.

To screen for mutations in the activation segment (exon 15) of the *B-raf *kinase domain, 100 ng of each melanoma DNA sample were PCR amplified by primers B-raf-6998-for and B-raf-70221-rev in 25 μl PCRs as described above. 5 μl aliquots of each reaction were electrophoresed on 1.5% agarose/ethidiumbromide gels using highly resolving NuSieve 3:1 agarose (Biozym). Positive controls were generated by a primer-mediated PCR mutagenesis-based protocol as described (Ruiz *et al*, 1997). Here, we incorporated a third longer primer, B-raf-70009-A-for or B-raf-70006-T-for, into the PCR along with the two wild-type primers B-raf-6998-for and B-raf-70221-rev. Longer mismatched primers (LMP) share the sequence of the wild-type primers but also contain additional bases, which include a mismatched base. Primer sequences according to the database of the National Center for Biotechnology Information (NCBI, Bethesda, MD, USA, ) were: A-Myb-1, 5'-CAT ggA ATg CCA ATT TAA Cg-3'; A-Myb-2, 5'-CAT CCC TAA gTT CgC TgC C-3' (gene database accession number X66087); B-raf-6998-for, 5'-ggC CAA AAA TTT AAT Cag Tgg A-3' (identical with primer sequence exon-15-rev according to [3]); B-raf-70221-rev, 5'-TCA TAA TgC TTg CTC TgA TAg gA-3' (identical with primer sequence exon-15-for according to [3]; gene database accession number NM_004333); B-raf-70009-A-for, 5'-ggC CAA AAA TTA AAT Cag Tgg A-3'; B-raf-70006-T-for, 5'-ggC CAA AAT TTT AAT Cag Tgg A-3'.

### SSCP gel electrophoresis

The amplified DNA was mixed with an equal volume of formamide loading dye (94% formamide, 0.05% xylene cyanol and 0.05% bromphenol blue), denatured at 95°C for 5 min, chilled on ice for 1 min and loaded onto Gene Excel 12.5/24 polyacrylamide gels (Pharmacia Biotech, Freiburg, Germany). Following non-denaturing electrophoresis at 600 Volt, 25 mA, 15 W, 6°C for 80 min, DNA fragments were stained by silver using the DNA silver staining kit according to the manufacturer's protocol (Pharmacia Biotech). DNA fragments that reproducibly showed mobility shifts according to independent two-repeated SSCP analyses were cut out from the acrylamide gel and subjected to semi-nested PCRs performed with primers B-raf-70023-for and B-raf-70221-rev using the conditions described above. Sequence of primer B-raf-70023-for was: 5'-ATA gCC TCA ATT CTT ACC ATC C-3'.

### Cloning of PCR amplicons and sequencing of plasmid inserts

Nested PCR products were then purified using the QIAquick PCR purification kit (QIAGEN) and cloned into pCR^R^2.1-TOPO vector (Invitrogen, NV Leek, Netherlands) according to the manufacturer's instructions. Briefly, 10 ng of the secondary PCR product were ligated into 10 ng vector and the ligation mixture was introduced into competent TOP10 bacteria by heat shock. The library was plated onto LB plates containing 50 μg/ml ampicillin. Single bacterial transformants that appeared positive in blue/white screening were picked randomly and grown in 2 ml LB medium containing 50 μg/ml ampicillin at 37°C overnight. Following alkaline lysis of bacterial cultures neutralized lysates were loaded onto silica-gel membranes (QIAprep spin miniprep kit, Qiagen) and plasmid DNAs were eluted in low-salt buffer.

Plasmid inserts were sequenced at a concentration of 50 ng/μl with a GeneAmp PCR system 9600 using ABI Prism dGTP BigDye Terminator Ready Reaction Kits and the AmpliTaq DNA polymerase FS according to the manufacturer's protocol (Perkin Elmer; Seqlab, Göttingen, Germany). For sequencing *B-raf *exon 15 fragments from both sides, M13 forward and M13 reverse primers were used. PCRs consisted of 25 cycles including a denaturation step at 96°C for 10 sec, a primer annealing step at 50°C for 5 sec and a chain elongation step at 60°C for 60 sec. Cycle sequencing products were then ethanol precipitated, run on a 4% polyacrylamide 7 M urea gel and analysed with the ABI Prism 377 Genetic Analyser (Perkin-Elmer; Seqlab). Primer sequences were: M13 forward primer, 5'-CAA AAg ggT CAg TgC Tg-3'; M13 reverse primer, 5'-gTC CTT TgT CgA TAC Tg-3'. The resulting sequences were aligned to the known *B-raf *sequence in the NCBI database (accession number NM_004333). The numbering began with the start codon ATG, corresponding to nucleotide positions 1–3. B-raf protein sequences predicted by the cDNA sequences were compared with B-raf wild type protein sequence (NCBI accession number NP_004324) using the BLASTX software at the NCBI.

### Statistical Analysis

Fisher's exact test was used to assess clinical data and histologic characteristics of the preceding primary melanomas as possible prognostic factors for the occurance of *B-raf *exon 15 mutations and to compare the results in cutaneous/subcutaneous melanoma metastases with data very recently reported from primary melanoma resection specimens [6]. The exact Wilcoxon rank sum test [10] was applied to test the hypothesis of *B-raf *exon 15 mutations being associated with tumour thickness according to Breslow in the preceding primary melanomas. Length of time between excision of primary melanomas and the occurance of cutaneous/subcutaneous metastases which we investigated was compared with length of time between excision of the cutaneous/subcutaneous metastases that we analysed and subsequent metastases by the exact Wilcoxon signed rank test. For all statistical analyses the statistical software package R, version 1.7 [11], was used. All statistical tests were 2-sided. An effect was considered statistically significant at P < 5%.

## Results

### Pattern of *B-raf *exon 15 mutations

**Figure 1:**
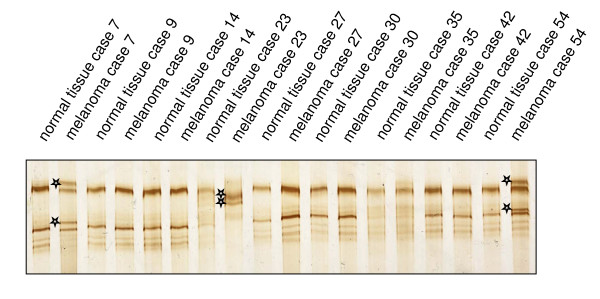
**PCR-SSCP analysis of *B-raf *exon 15 in resection specimens from cutaneous/subcutaneous melanoma metastases. **Cases 7, 23, and 54 exhibit different DNA fragment mobilities when compared to the respective normal tissues from patients. Suspecting these tumour specimens to harbour mutations, these samples were subjected to DNA sequencing.

Assessing the presence of amplifyable DNA by PCR amplification of a 184 bp human *A-myb *gene fragment, 60 resection specimens of melanoma cutaneous/subcutaneous metastases were positive for both cancerous and matched noncancerous tissues. Positive results in *A-myb *PCR were in accordance with agarose gel electrophoresis showing the integrity of the high molecular weight DNA. These melanoma resections specimens were then analysed by PCR and SSCP gel electrophoresis for mutations in exon 15 of the *B-raf *gene. 24/60 (40%) cases exhibited SSCP patterns distinctly different obtained from corresponding adjacent normal tissues (Figure 1). The total cellular DNA samples amplified with elongated primers that harboured mutations were identified as positive controls in all PCR-SSCP analyses (data not shown).

**Figure 2:**
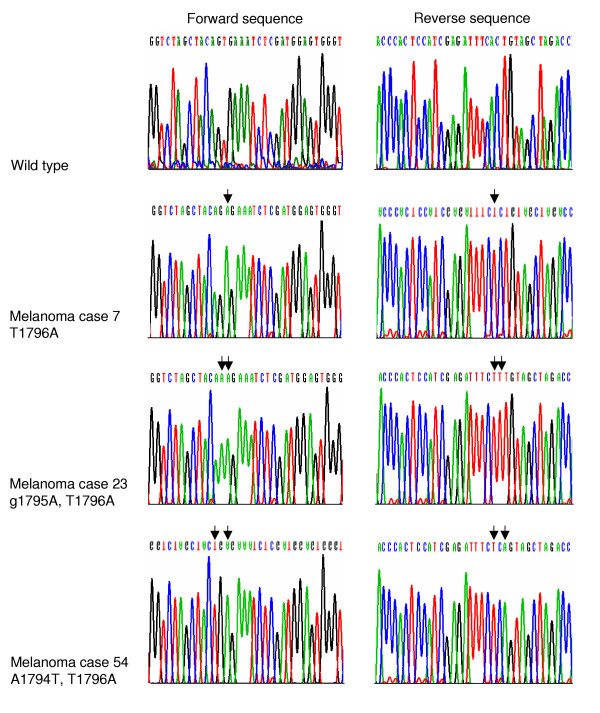
**Part of the DNA sequence of the activation segment of the *B-raf *kinase domain. **Representative melanoma resection specimens are shown which harbour missense mutations. In comparison to wild type DNA sequence (upper electropherogram, forward sequence on the left, reverse sequence on the right), melanoma cases 7, 23 and 54 exhibit nucleotide transitions, among them the activating T1796A mutation.

Sequencing of cloned PCR-SSCP amplicons resulted in 24 positive melanoma samples harbouring a total of 44 DNA point mutations of *B-raf *exon 15 (Table 1). The activating mutation T1796A [3] was present in 24 (40%) of the 60 investigated melanoma metastases (Figure 2), followed in frequency by the g1795A mutation in 8 cases (13%). Altogether, 16 different *B-raf *exon 15 mutations (T1749C, g1757A, C1758A, T1760C, T1779C, T1787A, A1794T, g1795A, T1796A, T1803g, A1810g, A1823g, g1824A, A1830C, T1837C, T1847C) were detected in the 60 investigated cutaneous melanoma resection specimens. In all melanomas, the mutations in neoplastic tissues were shown to be somatic by cloning and sequencing of SSCP gel bands from the corresponding normal tissues.

It is remarkable that CC to TT or C to T transitions, which occur in the *p53 *gene in non-melanoma skin cancers following exposure to ultraviolet light [12-15] were not detected in any of the investigated melanoma resection specimens.

### Predicted changes of the B-raf protein sequence

Next, the B-raf protein sequences predicted by the nucleotide sequences that we found were compared with the known B-raf protein sequence (NCBI accession number NP_004324) using the BLASTX software. All melanomas harbouring *B-raf *point mutations were predicted to exhibit alterations of the protein sequence (Table 1). Within the kinase domain, the oncogenic V599E amino acid substitution occurred in 19 of the 60 tested melanomas (32%), followed in frequency by V599K in 6/60 (10%) cases, both protein mutations occurring in two separate DNA fragments from melanoma case 46 (Table 1). Valine at protein sequence position 599 was replaced in all (100%) of the melanoma resection specimens positive for *B-raf *exon 15 mutations. No nonsense mutation of *B-raf *exon 15 DNA, causing a predicted protein truncation, occurred in our analysis.

Altogether, 9 different *B-raf *exon 15 protein mutations (D586N, L587P, F594L, V599E, V599K, S604G, E610D, S613P, I616T) were detected in the 60 investigated cutaneous/subcutaneous melanoma resection specimens. In comparison to the 16 different protein mutations very recently described in 19/50 *B-raf *mutations bearing primary melanomas [6], a narrowing of the spectrum of predicted B-raf protein mutations is observed during transition from primary melanomas towards cutaneous/subcutaneous metastases (p = 0.04 upon Fisher's exact test; estimated odds ratio 2.64, 95% confidence interval 0.97–7.63).

Applying Fishers's exact test, Clark's level of the primary melanoma (p = 0.46 and p = 0.55, respectively), level of UV irradiation of the preceding primary tumours (localization of the primary melanomas with chronic, intermittent or none UV exposition, p = 0.72 and p = 0.54, respectively) and type of subsequent metastasis (none, cutaneous, lymph node, visceral; p = 0.52 and p = 0.75, respectively) were no statistically significant prognostic factors for the occurence of valine substitution at B-raf amino acid position 599 (V599) or for the detection of the oncogenic V599E mutation, respectively. The occurence of valine substitution at B-raf amino acid position 599 (V599) or the detection of the V599E mutation were not associated with a change in the distribution of tumour thickness according to Breslow of the prededing primary melanomas (exact Wilcoxon rank sum test: p = 0.40 and p = 0.46, respectively). Unexpectedly, the histologic diagnosis of acrolentiginous melanoma in the preceding primary tumour (Fishers's exact test: p = 0.02 and 0.04, respectively) was associated with the absence of V599 and V599E in the cutaneous/subcutaneous metastases.

### *B-raf *mutations and clinical outcome

56 of the 60 patients were seen in follow up examinations. Following cutaneous/subcutaneous metastases, subsequent metastasis occurred in 55 patients including 11 cutaneous, 9 lymph node and 35 visceral decays (lung 15, bone 7, liver 6, brain 6, kidney 1). The median follow up period for all patients was 17 months (lower quartile 5.75 months; upper quartile 25 months).

Addressing the 11 patients with only subsequent cutaneous metastasis and the single patient which remained free of disease for 50 months (case 18), the follow up period of this subgroup was longer with a median of 22 months (lower quartile 17.25 months; upper quartile 25.25 months). The time between excision of primary melanomas and the occurance of cutaneous/subcutaneous metastases which we investigated (median 51 months; lower quartile 20 months; upper quartile 111.5 months) was longer in comparison to the length of time between excision of the cutaneous/subcutaneous metastases that we analysed and subsequent metastasis during follow up (exact Wilcoxon signed rank test: p < 0.001 ; estimated median difference 49 months with 95% confidence interval ranging from 29 months to 70.5 months). Following a long period free of disease after excision of the primary tumour, cutaneous/subcutaneous metastasis may therefore be regarded as a first indicator of melanoma progression, shortly followed by subsequent visceral metastasis in most patients.

Looking at the 24 cutaneous/subcutaneous melanoma metastases harbouring the oncogenic V599E or V599K amino acid substitutions, 21 patients were seen in follow up examinations and all of them developed subsequent metastasis. In comparison, 35/36 patients with *B-raf *exon 15 wild type metastases were monitored in follow up examinations from which 34 developed further metastasis. A single patient (melanoma metastasis case 18) remained free of tumour in a follow up interval of 50 months.

Comparing mutations of *B-raf *exon 15 in cutaneous/subcutaneous metastases with those in primary melanomas [6] by Fisher's exact test, no statistically significant differences in the proportion of mutations in general (p = 0.85; odds ratio = 0.92, 95% confidence interval 0.39–2.13), T1796A (p = 0.56; odds ratio = 0.77, 95% confidence interval 0.33–1.81), V599 (p = 0.56; odds ratio = 0.77, 95% confidence interval 0.33–1.81), V599E (p = 0.40; odds ratio = 0.68, 95% confidence interval 0.26–1.72) and V599K (p = 0.77; odds ratio = 1.22, 95% confidence interval 0.30–4.94) could be observed.

## Discussion

Cancers arise owing to the accumulation of mutations in critical genes that alter normal programmes of cell proliferation, differentiation and death. One important pathway mediating cellular responses to growth signals is the RAS-RAF-mitogen-activated protein (MAP)-kinase kinase (MEK)-extracellular signal-regulated kinase (ERK)-MAP kinase cascade whose activation can be achieved by mutation at various levels [16]. *RAS *is mutated to an oncogenic form in about 15% of human cancer. In a mouse model null for the tumour suppressor *INK4a*, melanoma initiation and maintanance were dependent upon expression of H-Ras^V12G ^[17]. Still, *H-Ras *as well as *K-ras *mutations rarely occur in human cutaneous melanomas [18,19] and are even less frequent in comparison to *N-Ras *which is mutated in less than 15% of uncultured melanoma tissue specimens [19-22]. Neither quantitative nor qualitative alterations of ras-p21 expression were found to correlate with tumour progression [20]. Downstream of Ras and upstream of MEK lies B-raf, which is highly present in neural cells and tissues [23-25]. This kinase belongs to the Raf family of serine/threonine kinases regulated by binding RAS which is composed of the ubiquitously expressed Raf-1 and by A-Raf and B-raf [16]. RAF proteins phosphorylate MEK1/2, which in turn phosphorylate ERK1/2. Very recently, *B-raf *somatic missense mutations have been reported in 59% of 34 melanoma cell lines [3]. All mutations were within the kinase domain, with a single substitution (V599E) accounting for 95% of all B-raf mutations in the melanoma cell lines.

In preliminary series of primary melanomas, 5/9 [3] and 4/5 [5] of the specimens harboured this V599E mutation, and only a single different mutation occurred in the *B-raf *oncogene outside of exon 15 [3,7]. Investigating a representative series of 50 resection specimens of primary cutaneous melanomas, we recently detected 12/50 cases (24%) to harbour the V599E transition independent from risk for further metastasis [6]. In contrast to cutaneous melanoma, V599E was detected in none of 46 familial melanomas [26], 21 multiple melanomas [26], 29 [27] and 48 [28] uveal melanomas. In 80 melanoma families, V599E was no germline mutation [29].

Addressing melanoma metastases, the V599E transition has recently been reported for 83% of 12 [5] and 50% of 28 [7] lymph node and in 64% of 12 [5] and 52% of 27 [7] visceral melanoma metastases. Looking at cutaneous/subcutaneous metastases, 63% of 29 cutaneous/subcutaenous cases were reported to harbour this oncogenic mutation [5]. These reports are in line with our data, the mutation rate in melanoma metastases similar to primary melanomas [6] suggesting V599E and V599K as early events in melanocyte transformation, possibly contributing to proliferation and survival, but not to metastatic spread. This postulate is supported by the finding of *B-raf *exon 15 mutations in a major subset of nevi [5,30].

As a novel finding, we here describe the spectrum of predicted B-raf protein mutations narrowing significantly during transition from primary melanomas towards cutaneous/subcutaneous metastases. Focusing on V599E and V599K in cutaneous/subcutaneous metastases, these oncogenic transitions are nonetheless not associated with enhanced risk for subsequent further metastasis. As the B-raf^V599E ^mutant possesses tenfold greater basal kinase activity and induces focus formation in NIH3T3 cells 138 times more efficiently than does wild-type B-raf [3], relevance of this alteration in the development of melanomas is very likely. For the growth of cancer cell lines with the V599E mutation, RAS function was not required any more [3]. The V599E mutation is thought to mimic phosphorylation of threonine 598 and serine 601 within the activation loop of *B-raf *at the plasma membrane, resulting in a protein with high activity and leading to constitutive ERK activation [31].

Whereas the missense mutation T1796A alone predicts the activating amino acid substitution V599E within the B-raf kinase domain, T1796A together with the g1795A nucleotide transition predict another acidic amino acid substitution, namely V599K with transforming activity in NIH3T3 cells comparable to V599E transfectants [32]. As a novel finding, we here describe the V599K mutation in 10% (6/60) of the tested cutaneous/subcutaneous metastases which is identical to the recently reported frequency of 10% (5/50) in primary melanomas [6]. In contrast to our data, V599K has been detected by other investigators in noticeable lower frequencies of 1% (1/77; [7]) and 3% (2/60; [30]) of melanoma metastases which may be due to higher sensitivity of the PCR-SSCP approach that we applied when compared to direct sequencing of tumour DNA samples.

As we found mutations within the activation segment (exon 15) of the B-raf kinase domain in a total of 24 (40%) of all investigated metastatic resection specimens which unexceptionally affected valine at protein sequence position 599, this protein sequence position seems to be a hot spot for alterations. The high frequency of *B-raf *mutations in melanomas may be related to a principal melanocyte-specific signalling pathway controlling proliferation and differentiation: α-melanocyte stimulating-hormone (α-MSH) and proopiomelanocortin-derived peptides, secreted by keratinocytes, bind to the melanocortin receptor I on melanocytes, leading to increased proliferation and melanogenesis in response to UVB radiation [33]. This signalling cascade via stimulation through G-protein coupled receptors (GPCRs) and upregulation of cyclic AMP (cAMP) does not require RAS but also activates B-raf and subsequently ERK [34]. When activated, extracellular signal-regulated kinases (ERKs) translocate to the nucleus where they regulate gene expression, leading to cell proliferation. The activation of ERKs by cAMP has been reported in a limited number of cell systems, including B16 melanoma [35].

Endothelin-1 (Et-1), a strong melanocyte mitogen [36,37], is another candidate for signaling through B-raf. Et-1, produced by keratinocytes and accentuated by exposition of keratinocytes to UVB radiation, can activate the MAP kinase pathway, although the role of B-raf in transducing this signal has not been demonstrated in melanocytes [38]. Besides α-MSH and Et-1, the RAS-RAF-MEK-MAP kinase cascade is also activated by the growth factors basic fibroblast growth factor [39] and stem cell factor [40], leading to increased proliferation of cultured human melanocytes.

That principal melanocyte-specific signalling pathways controlling proliferation and differentiation operate through activation of B-raf possibly explains the high frequency of *B-raf *mutations in melanomas when compared to colon (18% of 40 cell lines and 12% of 33 tumours) or ovarian cancers (4% of 26 cell lines and 14% of 35 tumours, according to [3]), an exception being papillary thyroid carcinomas with 69% (24/35 cases positive, [41]) and 36% (28/78 cases positive, [42]) of the investigated cases, respectively. The attempt of inhibiting B-raf activity in melanoma has been inspired by the demonstration that the ERK1/2 inhibitor U0126 [43,44] decreases proliferation of melanoma cell lines bearing *B-raf *mutations [3].

## Conclusion

Altogether, the serine/threonine kinase *B-raf*, involved in the Ras-Raf-MEK-ERK-MAP kinase pathway of signal transduction, harbours mutations of it's kinase domain in 40% of 60 melanoma metastatic resection specimens which is not exceeding the mutation frequency in primary melanomas [6] and which is not associated with enhanced risk for subsequent further metastasis. The oncogenic V599E and V599K transitions account for 79% and 25% of these positive metastases, respectively, one melanoma resection specimen (case 46) bearing both mutations. Acrolentiginous melanoma as preceding primary tumour was associated with the absence of V599 and V599E. During transition from primary melanomas towards cutaneous/subcutaneous metastases, the spectrum of predicted B-raf protein mutations narrows significantly. The *B-raf *mutations that we detect in uncultured primary melanoma samples are distinct from C to T or CC to TT mutations associated with pyrimidine dimer formation following UV radiation [12,13]. As B-raf alterations possibly affect melanocyte-specific pathways controlling proliferation and differentiation, inhibition of B-raf activity may be a future strategy in the treatment of melanoma as is currently addressed in phase I and II studies [45].

## Competing interests

The author(s) declare that they have no competing interests.

## Authors' contributions

Martin Deichmann designed the study and wrote the manuscript. Martin Deichmann and Marianne Thome carried out the PCR-SSCP analyses. Hjalmar Kurzen collected the melanomas and participated in DNA extraction. Axel Benner did the statistical analyses. Michael Kirschner and Judith Hassanzadeh participated in DNA cloning and DNA and protein sequence comparisons. All authors read and approved the final manuscript.

## Pre-publication history

The pre-publication history for this paper can be accessed here:



## Figures and Tables

**Table 1:** *B-raf *exon 15 mutations in resection specimens of melanoma cutaneous/subcutaneous metastases. Predicted protein sequence alterations are listed together with the clinical course of the disease in patients' follow up. Distinct mutations in two different abnormal bands in SSCP gels are specified by two lines for one melanoma.

Case	Previous primary (months before)	DNA mutations	Predicted Protein changes	Subsequent Metastasis (months later)
1	MM-2.7-IV (30 m)	T1796A	V599E	LN (1 m)
		g1757A	D586N	
7	NM-2.5-IV (11 m)	T1796A	V599E	liver (simult)
		A1810g	S604G	
12	SSM (18)	T1796A	V599E	liver (74 m)
13	NM-2.1-V (50)	T1796A	V599E	liver (2 m)
20	NM-4-IV (51)	T1796A	V599E	none (74 m)
		T1837C	S613P	
22	MM (19)	T1796A	V599E	sc (7 m)
23	MM-1.9-III (37)	g1795A, T1796A	V599K	lung, liver (simult)
24	MM-2.5-IV (84)	T1796A	V599E	bone (simult)
28	no data	g1795A, T1796A	V599K	no follow up
		C1758A, T1760C, g1795A, T1796A	V599K, L587P	
29	NM-3.6-IV (13)	g1795A, T1796A	V599K	liver (3 m)
32	no data	g1795A, T1796A	V599K	no follow up
		g1795A, T1796A, T1803G	V599K	
34	no data	T1779C, T1796A	F594L, V599E	no follow up
37	SSM-1.8-IV (120)	T1796A	V599E	sc (2 m)
		T1796A, A1823g	V599E	
41	MM (144)	T1796A	V599E	lung (simult)
43	SSM-1.8-IV (21)	T1796A	V599E	lung (3 m)
44	MM (71)	T1796A	V599E	lung (simult)
46	LMM-2.7-IV (53)	T1796A	V599E	bone (20 m)
		g1795A, T1796A	V599K	
47	MM-1.8-IV (126)	T1796A	V599E	none (42 m)
		T1787A, T1796A	V599E	
50	SSM-0.6-III (157)	T1749C, T1796A	V599E	brain, bone (simult)
		G1824A	WT	
53	no primary	T1796A	V599E	bone (simult)
		T1796A, T1847C	V599E, I616T	
54	LMM-III (290)	T1796A	V599E	sc (8 m)
		T1796A, A1794T	V599E	
55	MM-10-V (5)	T1796A, A1830C	V599E, E610D	lung (22 m)
56	MM (1)	T1796A	V599E	lung (1 m)
58	LMM-1.2-V (6)	g1795A, T1796A	V599K	sc (12 m)

**Abbreviations used in Table 1: **A, adenine; C, cytosine; D, asparagin acid; E, glutamic acid; F, phenylalanine; G, glycine; g, guanine; I, isoleucine; K, lysine; L, leucine; LMM, lentiginous malignant melanoma; LN, lymph node; m, month(s); MM-2.7-IV, melanoma, not further classified, tumour thickness according to Breslow 2.7 mm, Clark's level IV; N, asparagine; NM, nodular melanoma; P, proline; S, serine; sc, subcutaneous; simult, simultaneous; SSM, superficial spreading melanoma; T, thymine; T1857A, change from thymine to adenine at nucleotide 1857 (according to NCBI accession number NM_004333, the start codon ATG corresponding to nucleotide positions 1–3); V, valine; V599E, substitution of valine to glutamic acid at codon 599 (according to NCBI accession number NP_004324.1); WT, wild type of protein sequence.
